# Evidence of an epidemic spread of KPC-producing *Enterobacterales* in Czech hospitals

**DOI:** 10.1038/s41598-021-95285-z

**Published:** 2021-08-03

**Authors:** Lucie Kraftova, Marc Finianos, Vendula Studentova, Katerina Chudejova, Vladislav Jakubu, Helena Zemlickova, Costas C. Papagiannitsis, Ibrahim Bitar, Jaroslav Hrabak

**Affiliations:** 1 Department of Microbiology, Faculty of Medicine, and University Hospital in Pilsen, Charles University, Pilsen, Czech Republic; 2 Biomedical Center, Faculty of Medicine, Charles University, Pilsen, Czech Republic; 3 National Reference Laboratory for Antibiotics, National Institute of Public Health, Pilsen, Czech Republic; 4 Department of Microbiology, 3rd Faculty of Medicine, Charles University, University Hospital Kralovske Vinohrady and National Institute of Public Health, Prague, Czech Republic; 5 Department of Microbiology, University Hospital of Larissa, Larissa, Greece

**Keywords:** Antifungal agents, Antimicrobial resistance

## Abstract

The aim of the present study is to describe the ongoing spread of the KPC-producing strains, which is evolving to an epidemic in Czech hospitals. During the period of 2018–2019, a total of 108 KPC-producing *Enterobacterales* were recovered from 20 hospitals. Analysis of long-read sequencing data revealed the presence of several types of *bla*_KPC_-carrying plasmids; 19 out of 25 *bla*_KPC_-carrying plasmids could be assigned to R (n = 12), N (n = 5), C (n = 1) and P6 (n = 1) incompatibility (Inc) groups. Five of the remaining *bla*_KPC_-carrying plasmids were multireplicon, while one plasmid couldn’t be typed. Additionally, phylogenetic analysis confirmed the spread of *bla*_KPC_-carrying plasmids among different clones of diverse *Enterobacterales* species. Our findings demonstrated that the increased prevalence of KPC-producing isolates was due to plasmids spreading among different species. In some districts, the local dissemination of IncR and IncN plasmids was observed. Additionally, the ongoing evolution of *bla*_KPC_-carrying plasmids, through genetic rearrangements, favours the preservation and further dissemination of these mobile genetic elements. Therefore, the situation should be monitored, and immediate infection control should be implemented in hospitals reporting KPC-producing strains.

## Introduction

Carbapenem-resistant *Enterobacterales* (CRE) incidence have increased, causing worldwide public-health concern due to their rapid global dissemination and limited treatment options. Carbapenemases are enzymes able to hydrolyse almost all β-lactam antibiotics including carbapenems, one of the last drugs of choice. Carbapenemases are divided into different groups depending on their structure and hydrolytic activity^1^. *Klebsiella pneumoniae* carbapenemase (KPC) is the most predominant β-lactamase of class A carbapenemases. The KPC-type carbapenemases hydrolyse a wide variety of β-lactam antibiotics such as cephalosporins, penicillins and carbapenems^2^.

The *bla*_KPC_ gene was first identified in 1996 in North Carolina, USA, harboured by a *K. pneumoniae* isolate^3^. Later reports presented the monoclonal dissemination of KPC-producing isolates across America that was attributed to sequence type 258 (ST258) *K. pneumoniae*, as the predominant lineage^4^. Subsequently, KPC producers emerged in European countries, becoming highly endemic in some countries, especially in Greece and Italy^5, 6^. Other European countries had confirmed very few cases of *bla*_KPC_ up to 2013 according to the EuSCAPE project^7^. Even though KPC-type carbapenemases have been mostly associated with *K. pneumoniae* isolates, there are also reports of other bacterial species harbouring *bla*_KPC_-like genes, including *Escherichia coli*, *Citrobacter freundii*, *Klebsiella oxytoca* and other *Enterobacterales*, and *Pseudomonas aeruginosa*^8, 9^.

The *bla*_KPC_-like genes are most commonly found on the 10 kb transposon Tn*4401* and its isoforms^10, 11^. Until now, there are 9 isoforms of Tn*4401* (a to i)^12^. Due to the high mobilization efficiency of Tn*4401*, *bla*_KPC_-like genes have been identified on several plasmids belonging to different incompatibility (Inc) groups^13^.

In the Czech Republic, the first case of KPC-producing *K. pneumoniae* was identified in 2009^14^. This strain producing KPC-2, was collected from a patient repatriated from a hospital in Greece. Shortly after the first report, an outbreak of KPC-3-producing *K. pneumoniae*, belonging to ST512, was observed in another Czech hospital, with the index case being a patient repatriated from Italy. All those isolates harboured transposon isoform Tn*4401a*, carried on IncFII_K2_ pKpQIL-like plasmids^15^. Another ten KPC-2-producing *Enterobacterales*, mainly of the species *C. freundii*, were recovered in the University Hospital of Hradec Kralove (Czech Republic)^16^, during the period 2014–2016. Interestingly, sequencing revealed the presence of three plasmid types with the Tn*4401a* transposon. The first type comprised an IncR backbone and a KPC-2-encoding multidrug resistance (MDR) region, while the second type were derivatives of the first type carrying an IncN3-like segment. Finally, the third type was IncP6 plasmids sharing the same KPC-2-encoding MDR region with the two other types.

However, a significant increase in the number of KPC-producing isolates, referred to our laboratory from Czech hospitals, was observed since 2018. The aim of the present study is to describe the ongoing spread of the KPC-type producers, which is evolving to an epidemic in Czech hospitals, during the period of 2018–2019.

## Results

### KPC-producing *Enterobacterales*

During 2018–2019, a total of 490 *Enterobacterales* isolates with a meropenem MIC of > 0.125 μg/ml were referred to the National reference laboratory for antibiotics (Prague) or to the Biomedical Center (Pilsen) from 55 laboratories. All *bla*_KPC_-positive isolates (108) were subjected to further analysis described below. Distribution of laboratories is shown in Figure S1. Among them, 26 of the isolates were identified to be *K. pneumoniae*, 24 were identified to be *C. freundii*, 18 were identified to be *Enterobacter cloacae* complex, 14 were identified to be *Proteus mirabilis*, 11 were identified to be *Morganella morganii* and 10 were identified to be *E. coli*. The five remaining KPC-producing isolates belonged to the bacterial species, *Citrobacter farmeri* (n = 1), *Enterobacter aerogenes* (n = 1), *K. michiganensis* (n = 2) and *Klebsiella variicola* (n = 1) (Figure S2).

### Analysis of short-read sequencing results

Forty-nine out of 108 KPC producers, selected as representatives of all different hospitals, bacterial species and susceptibility profiles, were characterized by short-read sequencing using MiSeq (Illumina) platform. Based on short-read data, 44 of the 49 sequenced isolated harboured the *bla*_KPC-2_ allele (Table S1), while the five remaining isolates carried the *bla*_KPC-3_ gene. The *bla*_KPC-3_ allele was identified among 3 K*. pneumoniae*, 1 K*. michiganensis* and 1 *E. coli* isolates. Beside species-specific chromosomal β-lactamases, most of the clinical isolates also carried genes encoding OXA-1/9 oxacillinases (n = 37) and/or TEM-1 penicillinases (n = 34). The *bla*_CTX-M-15_ gene was found among 2 *Enterobacter* and 5 K*. pneumoniae* isolates, while 4 out of 5 *P. mirabilis* harboured the *bla*_CTX-M-14_ gene. Additionally, 4 out of 7 *Enterobacter* isolates co-carried the carbapenemase-encoding gene *bla*_VIM-4_. All sequenced isolates exhibited a wide variety of resistance genes conferring resistance to aminoglycosides, sulfonamides, trimethoprim, macrolides, streptogramin B, fosfomycin (low-level resistance), fluoroquinolones, chloramphenicol, tetracyclines, and/or rifampicin (Table S1).

WGS data revealed that *C. freundii* isolates belonged to sequence types ST65 (n = 6), ST580 (n = 3), ST98 (n = 2) and ST8 (n = 1) (Table S1). ST98 *C. freundii* isolates producing KPC-2 carbapenemase were previously recovered from critically ill patients hospitalized in Germany^17^, while ST8 *C. freundii* expressing a VIM-4 isoenzyme were identified in Poland^18^, in 2013. On the other hand, the novel ST580 was a single allele variant of ST142, which was previously associated with KPC-2 production in isolates from the University Hospital of Hradec Kralove (Czech Republic)^16^. The isolates belonging to *E. cloacae* complex were assigned to ST133 (n = 4) and ST421 (n = 3), which haven’t been previously associated with the production of KPC-2 carbapenemase. Additionally, in silico *hsp60* typing of the genome sequences showed that four *Enterobacter* isolates belonged to the species *Enterobacter hormaechei*^19^. The *K. pneumoniae* isolates included eight STs. Seven KPC-2-producers were distributed in ST101 (n = 4) and ST11 (n = 3). The remaining KPC-2-producing *K. pneumoniae* isolates belonged to unique STs (ST13, ST17 and ST147), while the *K. pneumoniae* isolates, which produced the KPC-3 enzyme, were ST307, ST512 and ST846. ST11, ST101, ST147, ST307 and ST512 have been previously associated with the spread of KPC resistance mechanism and have been considered as ‘high risk clones’^20, 21^. Finally, the *E. coli* and *K.* *michiganensis* (closely related to *K. oxytoca*) isolates were assigned to diverse STs, as shown in Table S1. Since MLST schemes do not exist for *M. morganii* and *P. mirabilis* isolates, phylogenetic clusters for the respective isolates were determined based on core-genome alignment (see below), using the Harvest suite^22^.

### Characterization of *bla*_KPC_-carrying genetic units

Based on short-read data, 25 KPC-producing isolates were selected to be sequenced by Sequel I platform, in an attempt to close plasmid sequences. All the 25 isolates showed resistance to cephalosporins and ertapenem while (except for *P. mirabilis* isolates) remained susceptible to colistin. Some variation in MIC values were noticed, however it is due to the different antibiotic resistance genes content found in each isolate (Table S3). Analysis of long-read sequencing data revealed the presence of several *bla*_KPC_-carrying plasmid sequences belonging to different Inc groups and presenting diverse sizes (Table S2). Based on PlasmidFinder analysis of plasmid sequences, 19 out of 25 *bla*_KPC_-carrying plasmids could be assigned tο R (n = 12), N (n = 5), C (n = 1) and P6 (n = 1) incompatibility (Inc) groups (Figure S3). Five of the remaining *bla*_KPC_-carrying plasmids were multireplicon, while one plasmid couldn’t be typed by the database. All plasmids, except IncN replicons, contained the Tn*4401a* isoform of the Tn*4401* transposon, which is similar to that described in plasmid pNYC, lacking 100 bp upstream of *bla*_KPC_ gene^11^.

**Figure 1:**
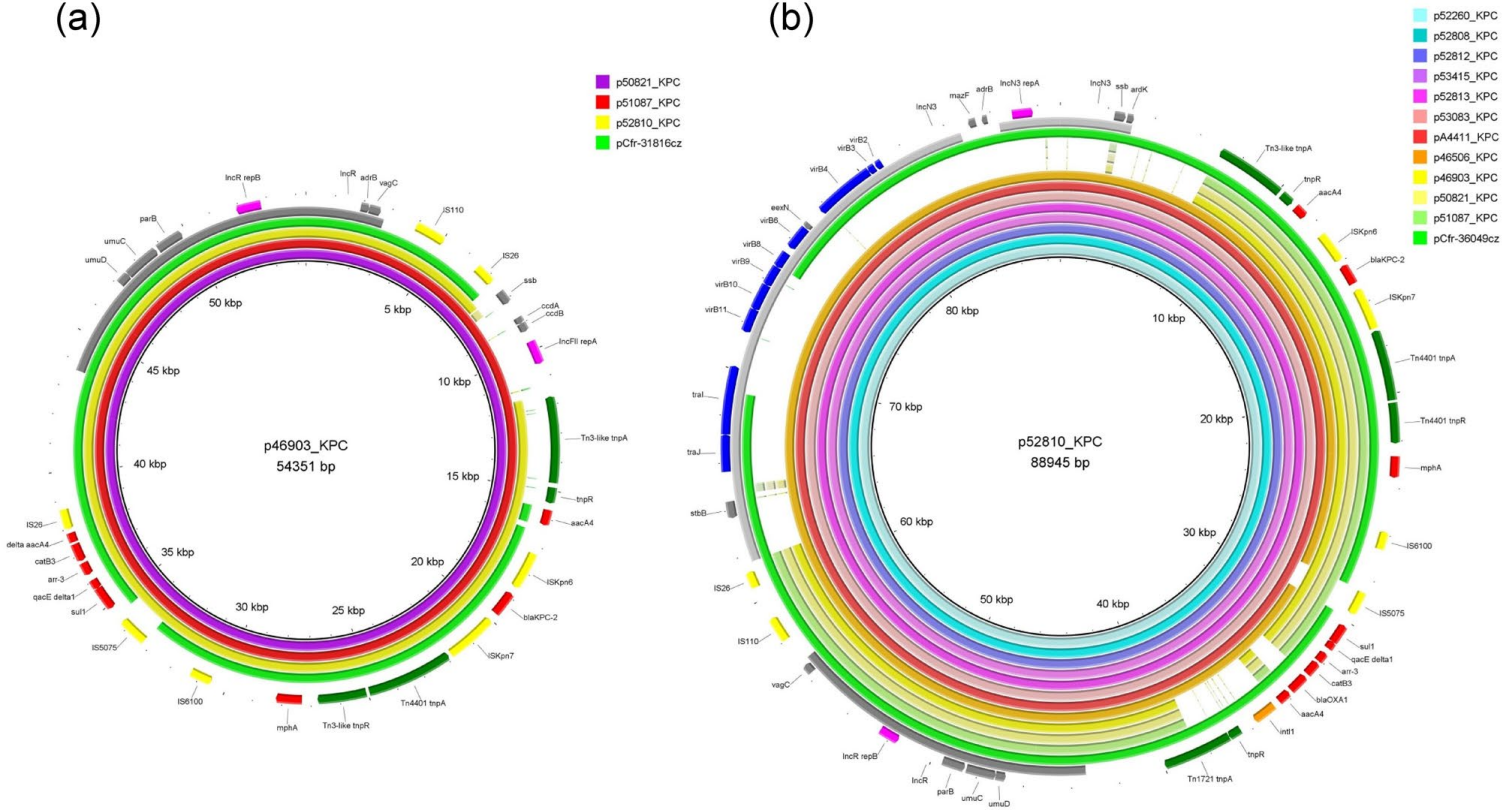
BRIG comparison of IncR KPC-encoding plasmids characterized from *Enterobacterales* isolates recovered from Czech hospitals.

Three out of 12 *bla*_KPC_-carrying plasmids, belonging to IncR group, were ⋍54 kb in size, while the nine remaining IncR plasmids sized ⋍89 kb. The IncR plasmids that were ⋍54 kb in size were derivatives of the IncR KPC-2-encoding plasmid pCfr-31816cz (Fig. 1a), which was characterized during an outbreak of KPC-2-producing *Enterobacterales* in a Czech hospital (Hradec Kralove)^16^. However, they differed from pCfr-31816cz by the presence of an additional 9232-bp sequence (nt 7286 to 16,517; GenBank accession no. CP070521) encoding CcdAB toxin-antitoxin system, and IncFIIA RepA and Ssb proteins. On the other hand, the IncR plasmids that were ⋍89 kb in size showed high degrees of similarity to each other and to the previously described plasmid pCfr-36049cz (Fig. 1b). Plasmid pCfr36049cz was characterized during the KPC-2 outbreak that took place in Hradec Kralove^16^, during 2014–2016. Similar to pCfr-36049cz, the latter plasmids were fusion derivatives of IncR *bla*_KPC-2_-positive plasmids and an IncN3-type-derived segment. However, unlike in pCfr-36049, a complete IncN3 transfer system was found, explaining the ability of pA4411_KPC, p46506_KPC, p52260_KPC, p52808_KPC, p52810_KPC, p52812_KPC, p52813_KPC, p53083_KPC and p53415_KPC to transfer via conjugation. Of note, the IncR plasmid p52813_KPC carried the *bla*_KPC-3_ allele, indicating the ongoing evolution of the determinants encoding KPC carbapenemases.

**Figure 2:**
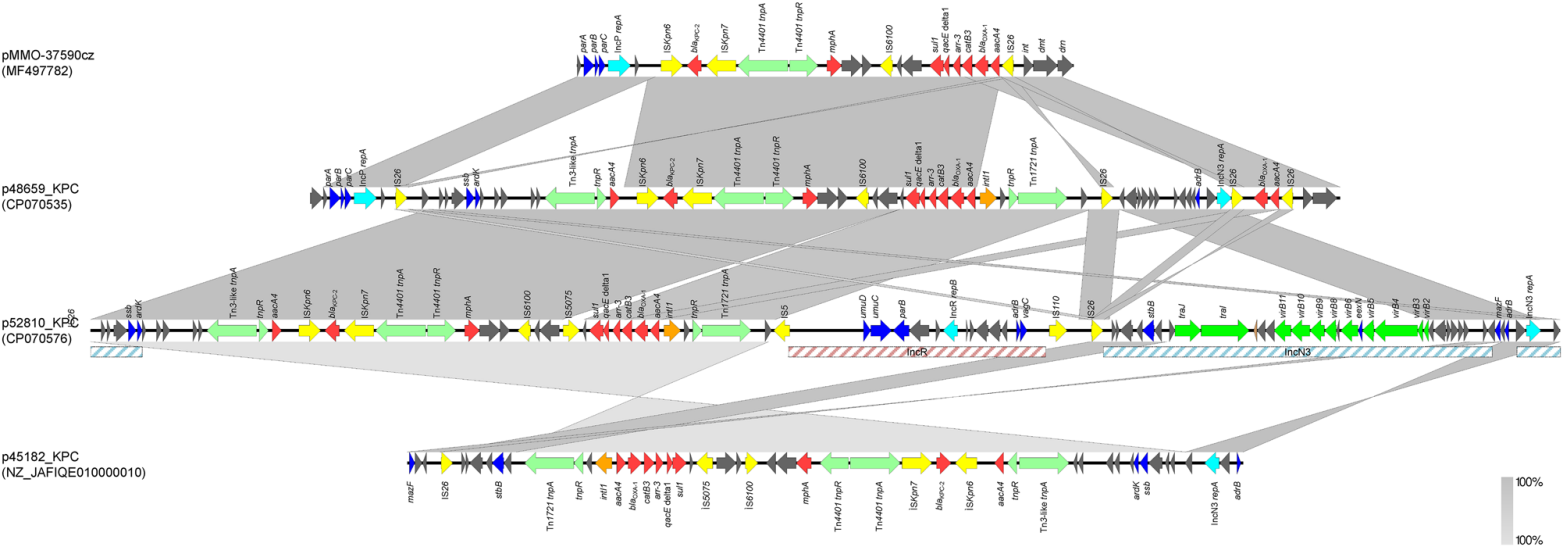
Linear comparisons of the KPC-encoding plasmids p48659_KPC and p45182_KPC. Arrows show the direction of transcription of open reading frames (ORFs). Resistance genes are shown in red. IS elements and transposases are shown in yellow and light green, respectively. *intI1* genes are shaded purple. Genes encoding replication, stability and transfer systems are shown in aqua, blue and green colors, respectively. The remaining genes are shown in gray. Homologous segments (representing ≥ 85% sequence identity) are indicated by gray shading.

Plasmid p48659_KPC is a fusion derivative of the p52810_KPC and pMmo-37590cz (Fig. 2). pMmo-37590cz is an IncP6 KPC-2-encoding plasmid that was also characterized during the KPC-2 outbreak in Hradec Kralove hospital^16^. Plasmid p48659_KPC contains a 50,603-bp sequence (nt 5917 to 56,519) encoding KPC-2, which is identical to a partial sequence of p52810_KPC. The remaining 11,723-bp sequence of p48659_KPC consists of one segment sharing extensive similarity with sequences carried by pMmo-37590cz. This segment included the IncP6 replication gene *repA*, the partitioning genes, *parA*, *parB*, and *parC*, and genes encoding a DNA invertase/recombinase (*int*), a deoxymethyltransferase (*dmt*), and a DNase (*drn*) of type II restriction module. Sequence analysis demonstrated that the plasmid p45182_KPC, which was not typed by PlasmidFinder, is 50,582 bp in size and is a derivative of p52810_KPC (Fig. 2). Only two differences between the two plasmids were observed. A 26,069-bp segment (nts 34,454 to 60,522 in p52810_KPC) including IncR plasmidic backbone, a Tn*1721*-like fragment (consisting of the 38-bp inverted repeat of the transposon, *tnpA*, *tnpR*, and *tnpM*), and *intI1* gene of the integron In*37*, was not present in p45182_KPC. In addition, a second fragment (nts 65,142 to 83,473 in p52810_KPC), being 18,332 bp in size, that contained *vir2/3/4/9/10/11* region of IncN3-like plasmids was also absent from p45182_KPC, probably explaining the inability of the plasmid to conjugate.

The IncN *bla*_KPC-2_-positive plasmids, which were assigned to ST15 based on the pMLST (https://cge.cbs.dtu.dk/services/pMLST/), comprised the plasmidic backbone and a multidrug resistance (MDR) region inserted downstream the *fipA*. The IncN plasmidic backbone contained a replication region (*repA*), a transfer system (*traA/B/C/D/N/E/O/F/G*), a stability operon (*stbA/B/C*) and an antirestriction system (*ardA/B*). The MDR region of IncN plasmids, which ranged from 21,011 to 31,420 bp in size, harboured a Tn*4401*-derived fragment of 2833-bp, encoding KPC-2 carbapenemase. In comparison to Tn*4401b*, the Tn*4401*-derived sequence (designated Tn*4401*j) had a deletion of 217 bp found upstream of the *bla*_KPC-2_. The Tn*4401*-derived fragment was disrupted by a Tn*3*-like sequence, 111 bp upstream of the *bla*_KPC-2_. The Tn*3*-like sequence was composed of the inverted repeat (IR) of the transposon and the *bla*_TEM-1_ resistance gene. A similar *bla*_KPC-2_-carrying genetic environment has been previously identified in the IncN plasmid pCF8698_KPC2, characterized from a *C. freundii* strain isolated in Germany (GenBank accession no. LN610760) (Figure S4). The MDR region of the IncN plasmids exhibited additional genes conferring resistance to aminoglycosides, sulfonamides, trimethoprim, macrolides, and/or fluoroquinolones (Table S2, Figure S4).

Plasmid p49969_KPC, typed as IncC based on PlasmidFinder, exhibited highest similarity with the OXA-204-encoding plasmid pCf3880 (74% coverage, 99.87% identity) (Figure S5). The pCf3880, which was an IncFII/FIB/C_2_ hybrid plasmid, was characterized from a *C.* *freundii* isolated from a hospital in Canada^23^. Unlike pCf3880, plasmid p49969_KPC carried the carbapenemase-encoding gene, *bla*_KPC-2_, which resulted from the acquisition of a 29,121-bp fragment (nt 111,204 to 140,234 in GenBank accession no. CP070549) showing extensive similarity to IncN plasmid p48846_KPC. The IncN-derived fragment was bound by two copies of the insertion sequence IS*26*, in parallel orientation, forming a composite transposon. Plasmid p49969_KPC carried, also, the resistance genes *bla*_TEM-1B_, *aac*(6')*-Im*, *aac*(3)*-IId* and *aph*(2'')*-Ib*.

**Figure 3:**
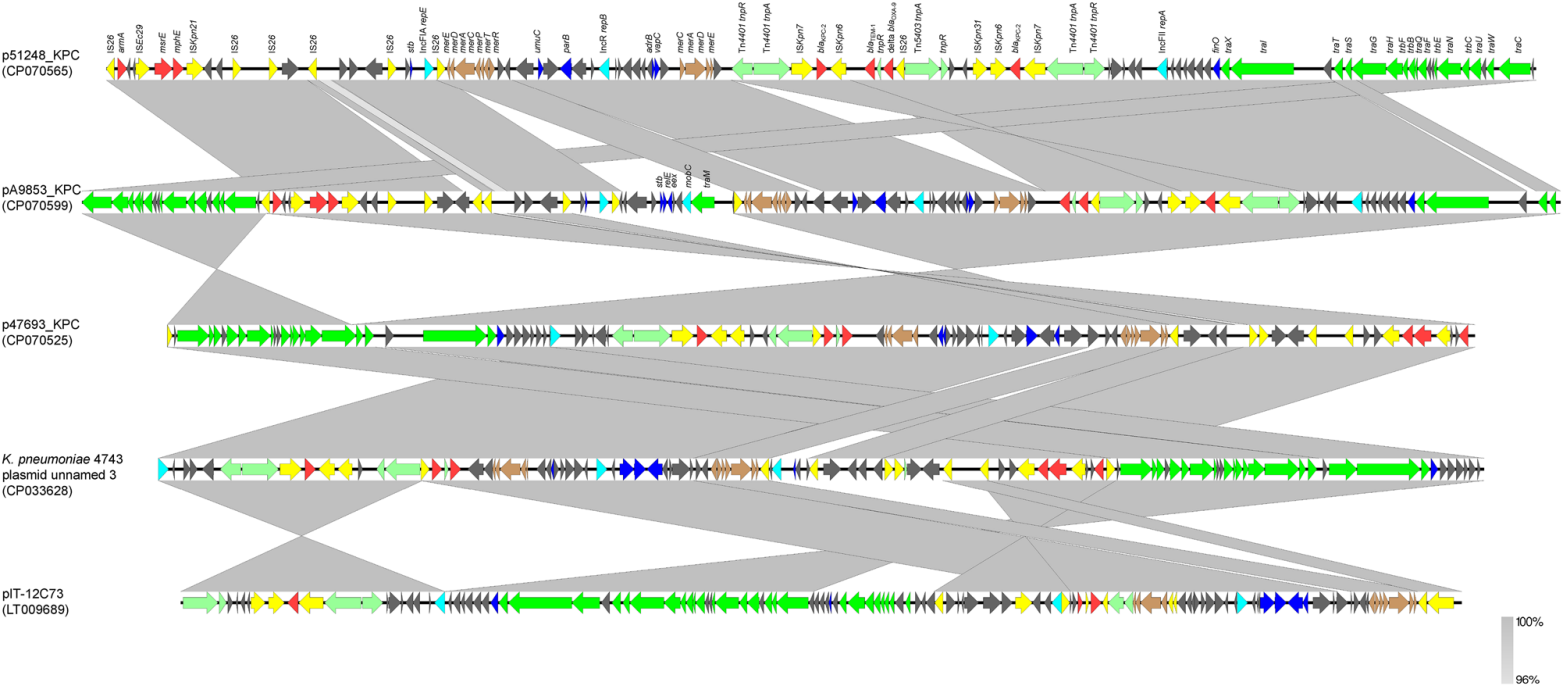
Linear comparisons of the multireplicon KPC-encoding plasmids p51248_KPC, pA9853_KPC and p47693_KPC. Arrows show the direction of transcription of open reading frames (ORFs). Resistance genes are shown in red, while genes involved in mercury resistance are shaded brown. IS elements and transposases are shown in yellow and green, respectively. *intI1* genes are shaded purple. Genes encoding replication, stability and transfer systems are shown in aqua, blue and green colors, respectively. The remaining genes are shown in gray. Homologous segments (representing ≥ 85% sequence identity) are indicated by gray shading.

Plasmids pA9853_KPC, p47693_KPC and p51248_KPC, which were characterized from ST101 *K. pneumoniae* isolates (Table S2), exhibited extensive similarity to pIT-12C73^20^ and plasmid unnamed 3 (GenBank accession no. CP033628) (Fig. 3). Plasmid pIT-12C73 which was also characterized from a ST101 *K. pneumoniae*, isolated in Italy in 2011, was a multireplicon IncFII_K2_-IncR KPC-encoding plasmid. Plasmids pA9853_KPC and p51248_KPC differed from pIT-12C73 by acquisition of a 3254-bp fragment containing the IncFIA *repE* gene. Plasmid pA9853_KPC harboured an additional 9295-bp sequence, being identical to ColE1-like plasmid pColRNAI-Kp1-1 (GenBank accession no. LC505458). Comparative analysis suggested that IS*26*-mediated recombination events likely played a major role in acquisition of fragments of diverse origin. Similarly, to pIT-12C73, apart from *bla*_KPC-2_, plasmids pA9853_KPC, p47693_KPC and p51248_KPC carried also *bla*_TEM-1_, *armA*, *mphE*, *msrE* resistance genes (Fig. 3). Interestingly, a duplication of the KPC-2-encoding transposon, Tn*4401a*, was found in plasmid p51428_KPC.

Plasmid p51483_ΚPC, which contained replicons FIB_K_ and FII_K_, showed highest similarity to KPC-2-encoding plasmid pRIVM_C008981_1 (96% coverage; 99.93% identity) (Figure S6a), characterized from a *K. pneumoniae* isolate collected in the Dutch national surveillance^24^. Similarly, to pRIVM_C008981_1, apart from regions responsible for conjugative transfer (*traX/I/D/T/S/G/H/F/B/Q/C/U/W/V/P/K/Y/M*) and plasmid maintenance (*psiA/B*, *parA/B* and *umuD/C* operons), p51483_ΚPC carried genes *silE/S/R/C/B/A/P* encoding a silver exporting ATPase, *pcoA/B/C/D/R/E* involved in copper resistance, *arsH/D/A/C/B/A/D/R* conferring arsenic resistance, and *fecI/R/A/B/C/D/E* implicated in Fe (3 +) dicitrate transport. In addition to *bla*_KPC-2_, plasmid p51483_ΚPC contained *bla*_TEM-1_, *aadA2*, *aph*(3')*-Ia*, *catA1*, *dfrA12* and *mphA* resistance genes. Finally, plasmid p51059_KPC, isolated from a ST512 *K. pneumoniae*, showed high similarity to IncFII_K2_ KPC-2 encoding plasmids pGR-1504 (99% coverage; 99.97% identity) and pIT-01C22 (coverage 99%; identity 99.96%) (Figure S6b) characterized from two endemic settings, Greece and Italy, during 2010–2011^20^. Plasmids pGR-1504 and pIT-01C22 were derivatives of the archetypal IncFII_K2_ KPC-encoding plasmid pKpQIL, originally described in the ST258 *K. pneumoniae* Kpn557 isolate^25^.

### Genomic comparison and relatedness

**Figure 4:**
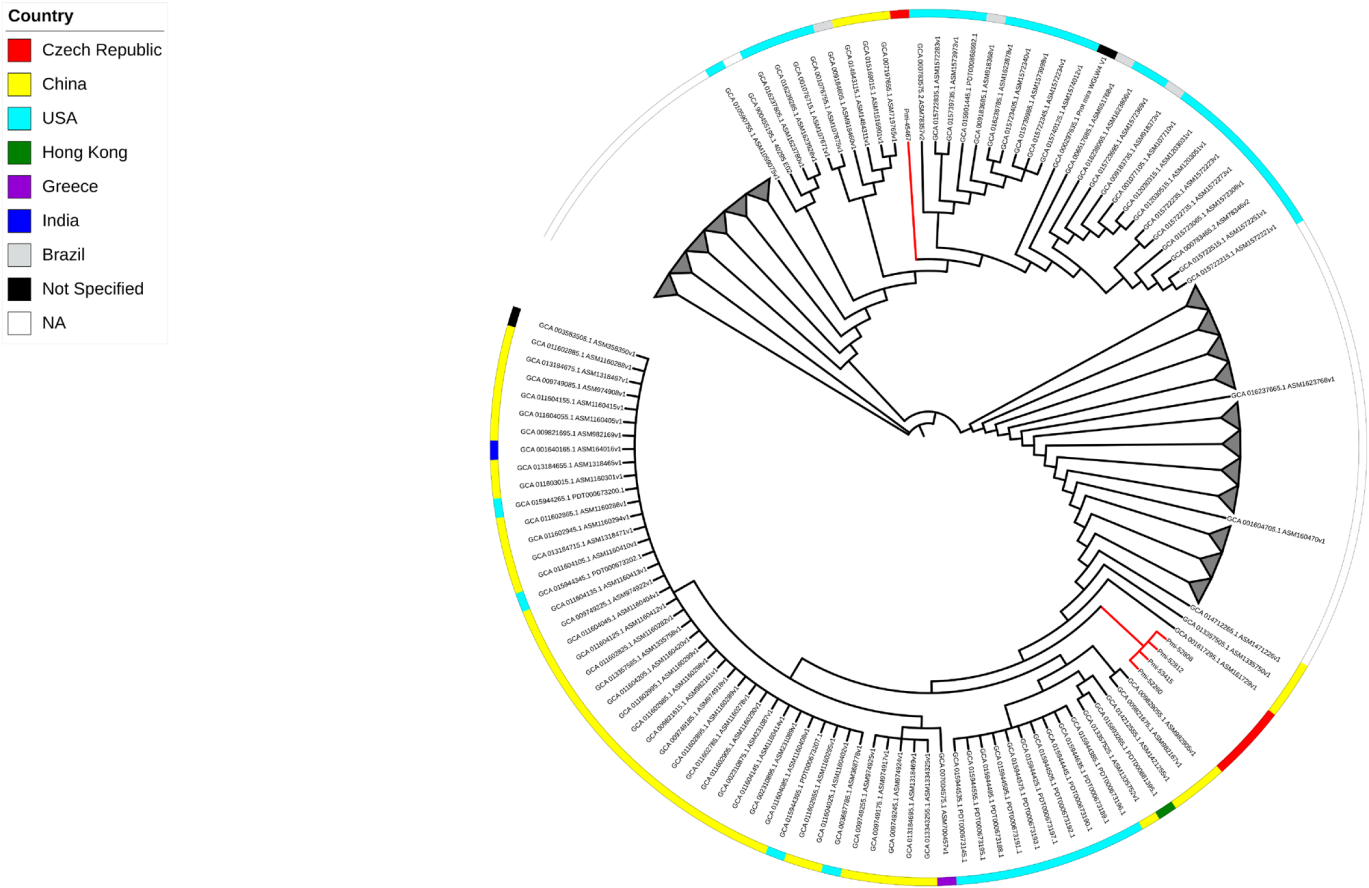
SNPs-based phylogeny of the five *P. mirabilis* isolates with 582 genomes downloaded from NCBI database. Red nodes indicate the Isolates from the study. Grey triangles indicate collapsed nodes.

Sequence data from the 49 sequenced isolates, have been used to investigate their genomic relatedness with global isolates and SNPs based phylogenies have been constructed accordingly. For *P. mirabilis*, the five sequenced isolates were compared against 582 genomes found in the NCBI database (Fig. 4). Four isolates (Pmi-52808, Pmi-52812, Pmi-53415 and Pmi-52260) clustered together forming a clade. These isolates were isolated from the same hospital (NY) and using Pmi-52260 as reference for SNPs detection, Pmi-52808 and Pmi-53415 shared 14 and 9 alterations (snps, del/ins), respectively, while Pmi-52812 shared 117 alterations (Table S4). Lastly, Pmi-45467, isolated in hospital HK, was relatively distant from the rest of the isolates.

**Figure 5:**
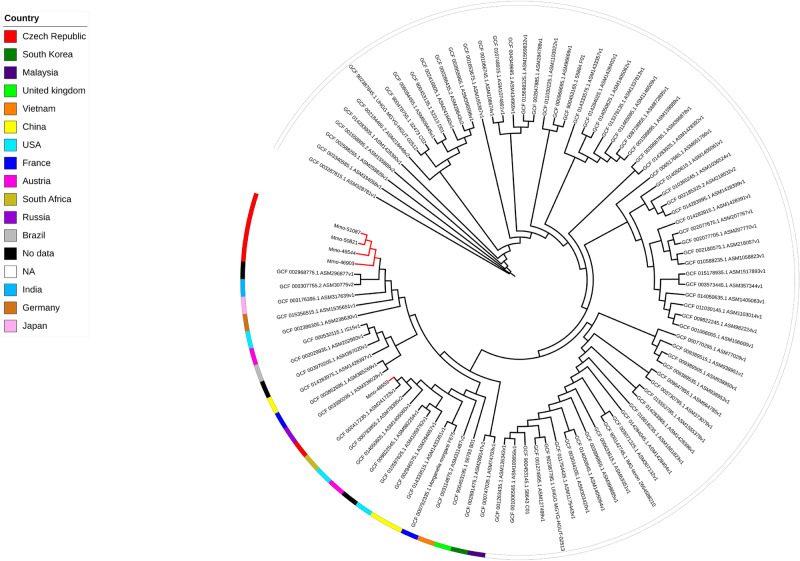
SNPs-based phylogeny of the five *M. morganii* with 92 genomes downloaded from NCBI database. Red nodes indicate the Isolates from the study.

Similarly, 92 M*. morganii* available genomes in NCBI database were downloaded to compare them with the five isolates sequenced in this study (Fig. 5). Mmo-48659, isolated in HK hospital, clustered alone in a unique node. However, it was closely related to an isolate from South Africa (790 alteration). On the other hand, four isolates (isolated from three different hospitals; Table S1) clustered together, with Mmo-51087 and Mmo-50821 isolated from the same hospital forming a subclade. For the detection of SNPs among the four isolates of this clade, Mmo-51087 was used as a reference. Mmo-50821 and Mmo-46544 showed 22 and 62 alterations, respectively, while Mmo-46903 222 alterations (Table S4).

**Figure 6:**
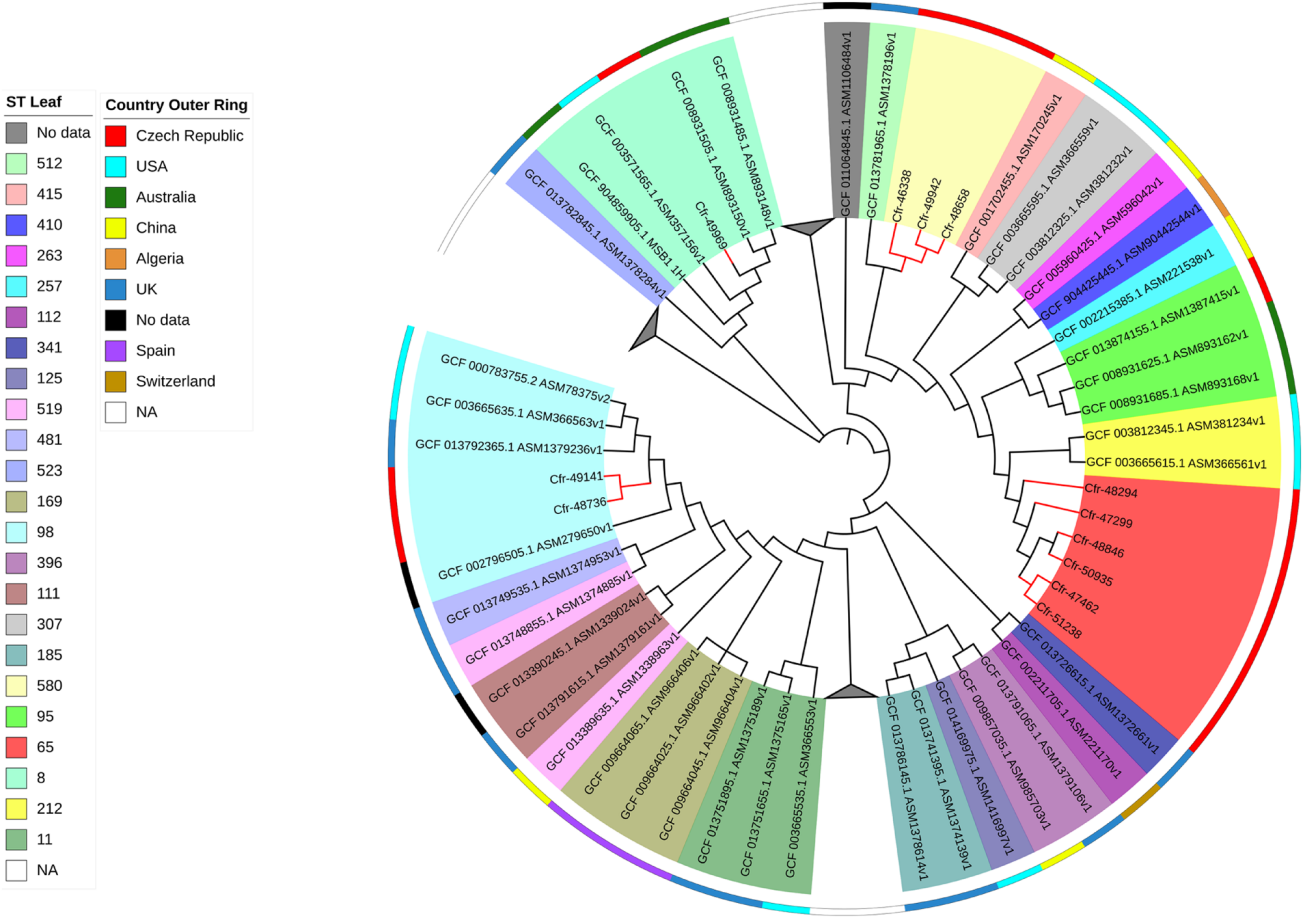
SNPs-based phylogeny of the 12 *C. freundii* with 118 genomes downloaded from NCBI database. Red nodes indicate the Isolates from the study. Grey triangles indicate collapsed nodes.

For *C. freundii*, 118 genomes were downloaded from the NCBI database to compare them with the 12 sequenced isolates (Fig. 6). The genomes of three isolates (Cfr-46338, Cfr-49942 and Cfr-48658), which belonged to ST580 and were recovered from HK hospital, clustered together forming a clade. SNPs detection among these isolates showed that Cfr-49942 and Cfr-46338 had 93 and 111 alterations, respectively, compared to Cfr-48658. In a closely related clade, another six genomes from ST65 isolates Cfr-50935, Cfr-48846, Cfr-51238, Cfr-47462, Cfr-48294 and Cfr-47299 clustered together. SNPs detection when compared to Cfr-50935 showed that Cfr-48846 had 25 alterations while Cfr-51238, Cfr-47462, Cfr-47299 and Cfr-48294 had 47, 57, 88 and 99 respectively (Table S4). On the other hand, the genomes of the two ST98 isolates, Cfr-48736 and Cfr-49141, clustered together in a considerable distant clade. These isolates are clustered together with other ST98 *C. freundii* isolates from the USA and UK. SNPs detection showed that Cfr-48736 had 28 alterations when compared to Cfr-49141. Finally, the isolate Cfr-49969, which was assigned to ST8, resulted in a unique node.

**Figure 7:**
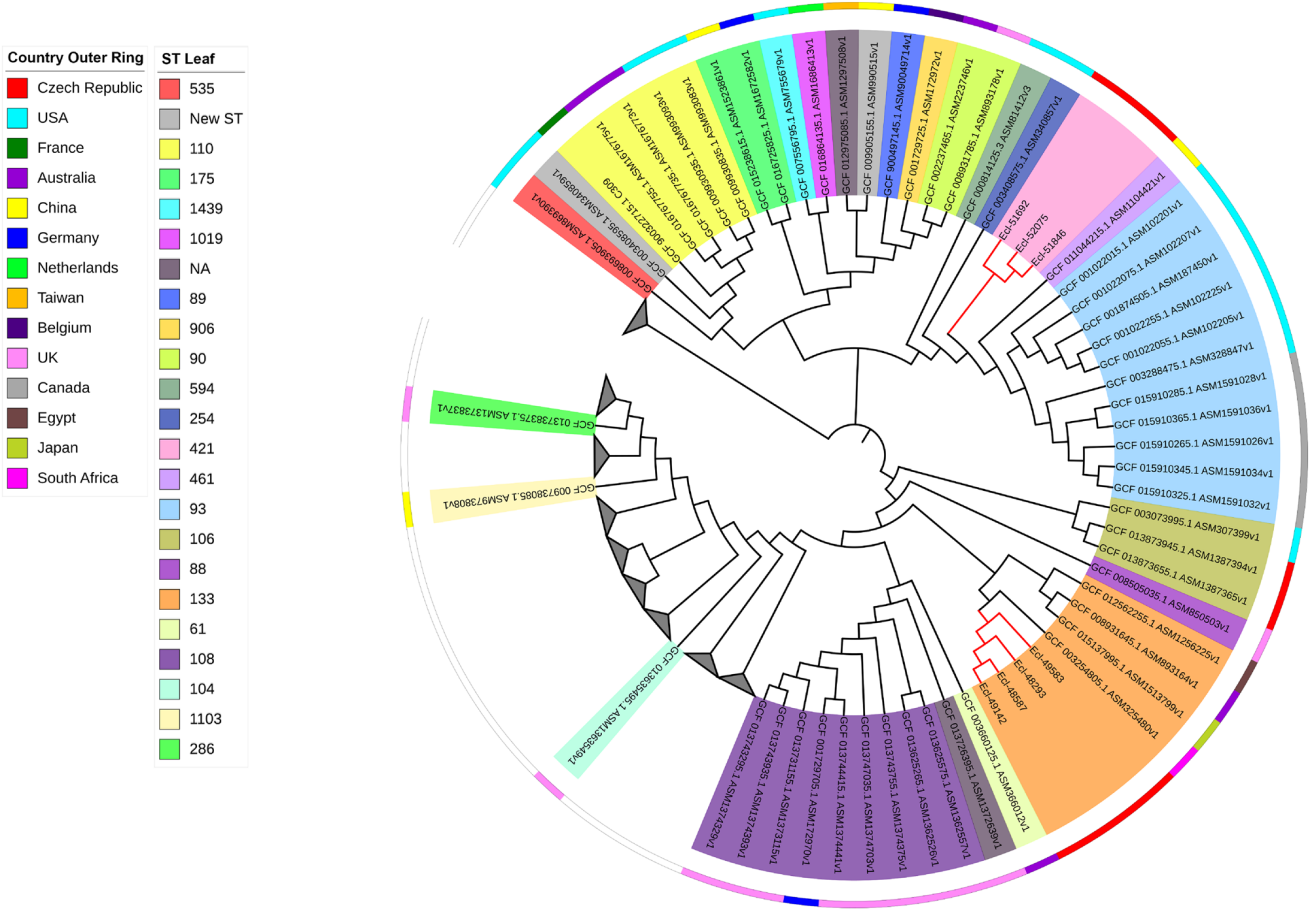
SNPs-based phylogeny of the seven *E. homaechei* with 126 genomes downloaded from NCBI database. Red nodes indicate the Isolates from the study. Grey triangles indicate collapsed nodes.

For *Enterobacter hormaechei*, 126 genomes were downloaded from the NCBI database and were compared with the seven isolates sequenced during this study (Fig. 7). The isolates clustered in two clades. The first clade contained four isolates (Ecl-49142, Ecl-48587, Ecl-48293 and Ecl-49583). Ecl-48293 was used as a reference genome for SNPs detection among these four isolates. Ecl-49142 had 26, Ecl-48587 had 34 and Ecl-49583 had 32 alterations. All ST133 isolates recovered from Czech Republic, South Africa, Japan, Australia and Egypt were grouped in a unique cluster. Additionally, the other cluster contained the three isolates (Ecl-51693, Ecl-51846 and Ecl-52075) which were ST421. For SNPs detection, Ecl-51846 was used as a reference, showing that Ecl-52075 and Ecl-51692 had 12 and 14 alterations, respectively (Table S4).

**Figure 8:**
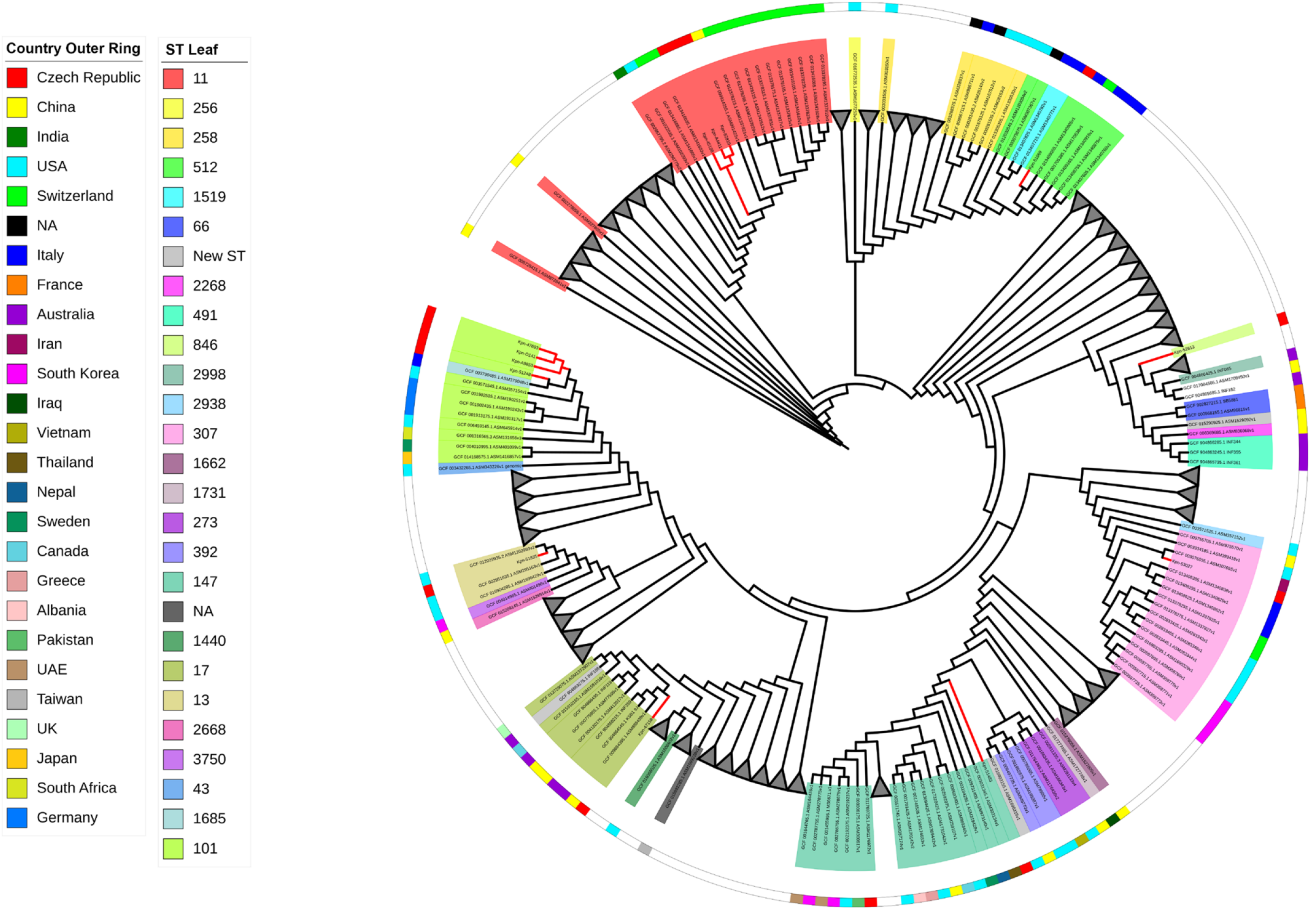
SNPs-based phylogeny of the 13 K*. pneumoniae* with 732 genomes downloaded from NCBI database. Red nodes indicate the Isolates from the study. Grey triangles indicate collapsed nodes.

For *K. pneumoniae*, 732 genomes were downloaded from the NCBI database to compare them with the 13 sequenced isolates (Fig. 8). Isolates Kpn-51835, Kpn-47158, Kpn-51483, Kpn-53027, Kpn-52813 and Kpn-51069, which were assigned to diverse STs, formed a unique distinct node each. One clade consisting of Kpn-47693, Kpn-O141 and Kpn-A9853 was in close proximity with Kpn-51248 in the neighbouring cluster. Using Kpn-47693 as a reference genome for SNPs detection, Kpn-O141, Kpn-A9853 and Kpn-51248 had 25, 22 and 100 alterations, respectively. The above isolates were grouped together with other ST101 isolates from Italy, USA, Japan, India and South Africa. The last three *K. pneumoniae* isolates (Kpn-52810, Kpn-A4411 and Kpn-45128) clustered together. Using Kpn-52810 as a reference genome for SNPs detection, Kpn-A4411 had 27 alterations, while Kpn-45128 exhibited 816 alterations (Table S4). Interestingly, the last isolates were clustered with isolates from China, Switzerland, India and the USA, which belonged also to ST11.

## Discussion

KPC-producing *Enterobacterales* represent a major threat of global dimensions for public health. The current study described the change of the epidemiological situation in Czech hospitals, from the sporadic cases or outbreaks to the epidemic spread of KPC-producing isolates (Figure S1). During 2018–2019, 108 KPC producers were recovered from 22 different Czech hospitals located throughout the country. Additionally, the *bla*_KPC_ gene was found among diverse species and clones of *Enterobacterales* family (Figure S2).

Phylogenetic analysis indicated that *P. mirabilis* and *M. morganii* isolates, carrying the *bla*_KPC_-like gene, didn’t exhibit close relationship with isolates characterized previously from other geographical areas. Additionally, phylogenetic analysis showed that the KPC-2-producing *E.* *hormaechei* isolates belonged to two distinct clones (Fig. 7), assigned as ST133 and ST421 based on MLST. The ST421 isolates weren’t closely related with other isolates analysed, using parsnp software, while ST133 isolates clustered together with isolates from South Africa, Japan, Australia and Egypt. However, ST133 isolates, recovered from other geographical areas, weren’t associated with the production of KPC-2 carbapenemase. Regarding *C. freundii* isolates, phylogenetic analysis revealed two main clones, which were assigned to ST65 and ST580 based on MLST. These two clones were distinct to each other and to the isolates included in the analysis. Isolates Cfr-48736 and Cfr-49141 grouped together with other ST98 isolates from the UK and USA, while the isolate Cfr-49969 was clustered with other ST8 isolates from Australia and the USA. However, the later clones have been associated with the production of KPC-2 and VIM-4 carbapenemases in Germany and Poland^17, 18^, respectively. On the other hand, 6 out of 13 K*. pneumoniae* isolates characterized by WGS belonged to unique STs. Interestingly, parsnp phylogenetic analysis clustered these isolates with other *K. pneumoniae* isolates, belonging to the same STs, from worldwide. Additionally, the 7 remaining *K. pneumoniae* isolates belonged to two distinct clades. The later clades included ST11 and ST101 isolates from different geographical origins. Among *K. pneumoniae*, the ‘high risk’ clones, ST11, ST101, ST147 and ST512, that have been previously associated with the spread of KPC resistance mechanism were found^20, 26^. In agreement with recent reports, those data confirm that high-risk clones, other than CC258, currently contribute to spread of KPC resistance mechanism in Europe^24, 25^. Finally, the KPC-producing *E. coli* and *K. michiganensis* isolates belonged to unique STs. These findings underline the ongoing spread of the KPC resistance mechanism among different species and clones.

The analysis of the genetic units carrying the *bla*_KPC_-like genes revealed the presence of a wide variety of plasmids involved in the spread of the KPC resistance mechanism. Some of the observed plasmid-types, like IncFII_K2_ pKpQIL, IncFII_K2_-IncR pIT-12C73, and IncR-IncN_3_ pCfr-36049cz, have been previously described to be responsible for the spread of the *bla*_KPC_-like genes^16, 20, 25^. Additionally, some novel emerging plasmid-types, as the IncN pCF8698_KPC2 originally described from Germany (GenBank accession no. CP070521), the IncFIB_K_/FII_K_ pRIVM_C008981_1 firstly characterized from a Dutch collection^24^, and the hybrid IncFII/FIB/C_2_/N plasmid p49969_KPC characterized during this study, were identified to disseminate the *bla*_KPC_-like genes. A few fusion derivatives of the *bla*_KPC_-carrying plasmids described above were observed. These data verify the presence of some successful plasmid lineages spreading the KPC resistance mechanism, but also highlight the ongoing evolution of the mobile genetic elements involved in the dissemination of clinically important resistance mechanisms. For example, IncR plasmids carrying *bla*_KPC_ genes have played a significant role in the spread of the specific resistance mechanism, in the Czech Republic. But, IncR plasmids have also been involved with the spread of other important carbapenemases, like NDM and VIM^27, 28^. Additionally, in agreement with previous studies^20, 25, 29^, IncF plasmids are one of the major factors contributing to the worldwide spread of KPC carbapenemases. Moreover, the distribution of the different plasmid types detected suggests local dissemination with IncR plasmid spreading in middle part of the map especially in Hradec Kralove and Nymburk (Figure S3), while the IncN plasmid spreading in the North West of the Bohemian region. However, Prague seems like the melting pot, in which all plasmid families were detected, indicating the transient admission of patients from surrounding districts to Prague for specialized treatment. Moreover, this is confirmed by the fact that most of the isolated strains in Prague comes from private labs which provides services for many hospitals and long-term care facilities in and outside Prague. This route of dissemination could be explained by the spread of specific plasmid families within the same region, like IncR plasmids, or crossing the borders via travelling, like IncN plasmid from Germany.

In conclusion, our results show that the increased prevalence of KPC-producing isolates was due to plasmids being conjugative and spreading among different species and clones. Additionally, the ongoing evolution through genetic rearrangements, observed in *bla*_KPC_-carrying plasmids, favour the preservation and further dissemination of these mobile genetic elements. Therefore, the situation should be monitored, and immediate infection control should be implemented in hospitals reported.

## Material and methods

### Bacterial isolates, carbapenemase production and susceptibility testing

From 2018 to 2019, National reference laboratory for antibiotics referred a total of 108 *Enterobacterales* isolates being PCR positive for *bla*_KPC_. Species identification was performed by matrix-assisted laser desorption ionization-time of flight mass spectrometry (MALDI-TOF MS) using MALDI Biotyper software (Bruker Daltonics, Bremen, Germany). All isolates were tested for carbapenemase production by the MALDI-TOF MS meropenem hydrolysis assay^30^. Additionally, the presence of carbapenemase-encoding genes (*bla*_KPC_, *bla*_VIM_, *bla*_IMP_, *bla*_NDM_, and *bla*_OXA−48_-like) was confirmed by PCR amplification^11, 31–33^. Antimicrobial susceptibility was performed using broth microdilution according to European Committee on Antimicrobial Susceptibility Testing (EUCAST) guidelines. Susceptibility data were interpreted according to the criteria (version v11.0) of the EUCAST (http://www.eucast.org/).

### Short-read whole genome sequencing

Forty-nine KPC-producing Enterobacterales were selected for complete sequencing, using the Illumina MiSeq platform (Illumina Inc., San Diego, CA, USA). These isolates were selected as representatives of all different hospitals, bacterial species and susceptibility profiles.

The genomic DNAs of the clinical isolates were extracted using the DNA-Sorb-B kit (Sacace Biotechnologies S.r.l., Como, Italy). Multiplexed DNA libraries were prepared using the Nextera XT library preparation kit, and 300-bp paired-end sequencing was performed on the Illumina MiSeq platform (Illumina Inc., San Diego, CA, USA) using the MiSeq v3 600-cycle reagent kit. Initial paired-end reads were quality trimmed using the Trimmomatic tool v0.33^34^ and then, assembled by use of the de Bruijn graph-based de novo assembler SPAdes v3.14.0^35^.

### Map

Maps of the Czech Republic was created using the Leaflet package^36^ in R-studio^37^ from R-project^38^.

### Long-read whole genome sequencing

Based on the results of short-read sequencing (see below), twenty-five KPC producers were selected to be sequenced using long-read sequencing technology, to help close the whole plasmid sequences. These isolates were selected as representatives of all different hospitals, bacterial species, STs, replicon profiles and KPC alleles.

Genomic DNA was extracted from the clinical isolates using NucleoSpin Microbial DNA kit (Macherey–Nagel, Germany). Whole genome sequencing (WGS) was performed on the Sequel I platform (Pacific biosciences, Menlo Park, CA, United States). Microbial multiplexing protocol was used for the library preparation according to the manufacturer instructions for Sheared DNA. DNA shearing was performed using the Megaruptor 2 (Diagenode, Liege, Belgium) using long hydropores producing 10 kb long inserts. No size selection was performed during the library preparation. The Microbial Assembly pipeline offered by the SMRT Link v9.0 software was used to perform the assembly and circularization with minimum seed coverage of 30X. Assembled sequences were annotated using the NCBI Prokaryotic Genome Annotation Pipeline (PGAP).

### Analysis of WGS data

Antibiotic resistant genes, plasmid replicons and multilocus sequence types (MLST) were determined through uploading the assembled sequences to ResFinder 4.1 and CARD^39, 40^, PlasmidFinder^41^, and MLST 2.0^42^, respectively.

For sequence analysis, the BLAST algorithm (www.ncbi.nlm.nih.gov/BLAST), the ISFinder database (www-is.biotoul.fr/), and open reading frame (ORF) finder tool (www.bioinformatics.org/sms/) were utilized. Comparative genome alignment was done using Mauve v.2.3.1. (http://darlinglab.org/mauve/mauve.html) and BLAST Ring Image Generator (BRIG)^43^. Diagrams and gene organization were sketched using Easyfig v.2.2.2^44^.

### Transfer of *bla*_KPC_-like genes

Conjugal transfer of *bla*_KPC_-like genes from the clinical strains was carried out in mixed broth cultures^45^, using the rifampicin-resistant *E. coli* A15 laboratory strain as a recipient. Transconjugants were selected on MacConkey agar plates supplemented with rifampicin (150 mg/l) and ampicillin (50 mg/l). Transconjugants were confirmed to be KPC producers by PCR^11^ and the MALDI-TOF MS meropenem hydrolysis assay^30^.

### Phylogenetic analysis

Genetic diversity and phylogenetic relationship between the sequenced samples and global genomes were studied. All phylogenies were created using core genome, recombination and single nucleotide polymorphisms (SNPs) using parsnp v1.2, available in the harvest suite^22^ using a corresponding reference genome. SNPs identified in local collinear blocks were subsequently used for reconstructing an approximate maximum-likelihood tree using FastTree2^46^ while including the general time reversible (GTR) model of nucleotide substitution. The Shimodaira–Hasegawa test implemented in FastTree2 was used to assess the support for significant clustering in the observed phylogeny. The interactive tree of life or iTOL (https://itol.embl.de/). ^47^ was used for the graphic illustration of the trees along with relative annotations.

For the construction of the SNPs-based phylogenies, 582 *Proteus mirabilis* genomes were downloaded from NCBI assembly database including complete and draft genomes, using ASM6996v1 as reference. Similarly, 92 genomes for *Morganella morganii* (ASM1428397v1 as reference)*,* 118 genomes for *Citrobacter freundii* (Cfr-49,969 as reference)*,* 126 genomes for *Enterobacter hormaechei* (Ecl-48,293 as reference) and 732 genomes for *K. pneumoniae* using Kpn-48,293 as reference.

Moreover, isolates from the study that clustered together forming a clade or/and subclade were investigated further. SNPs among the isolates within the clade/subclade were compared to a reference genome within the selected set using snippy v. 4.4.3 (https://github.com/tseemann/snippy).

### Nucleotide sequence accession numbers

The nucleotide sequence of the genomes and plasmids were deposited and available in GenBank under the BioProject number PRJNA700516; all accession numbers can be retrieved from Table S5.

## Supplementary Information

Supplementary Information 1.

Supplementary Information 2.

Supplementary Information 3.

Supplementary Information 4.

Supplementary Information 5.

Supplementary Information 6.

Supplementary Information 7.

Supplementary Information 8.

Supplementary Information 9.

Supplementary Information 10.

Supplementary Information 11.

Supplementary Information 12.
