# Supplementary material for: Evidence of an epidemic spread of KPC-producing Enterobacterales in Czech hospitals
Source: Sci Rep. 2021 Aug 3;11:15732. doi: 10.1038/s41598-021-95285-z (PMC8333104; doi:10.1038/s41598-021-95285-z)
Supplement: Supplementary file 2 — Supplementary Information 2. [file 41598_2021_95285_MOESM2_ESM.pptx]

## Slide 1
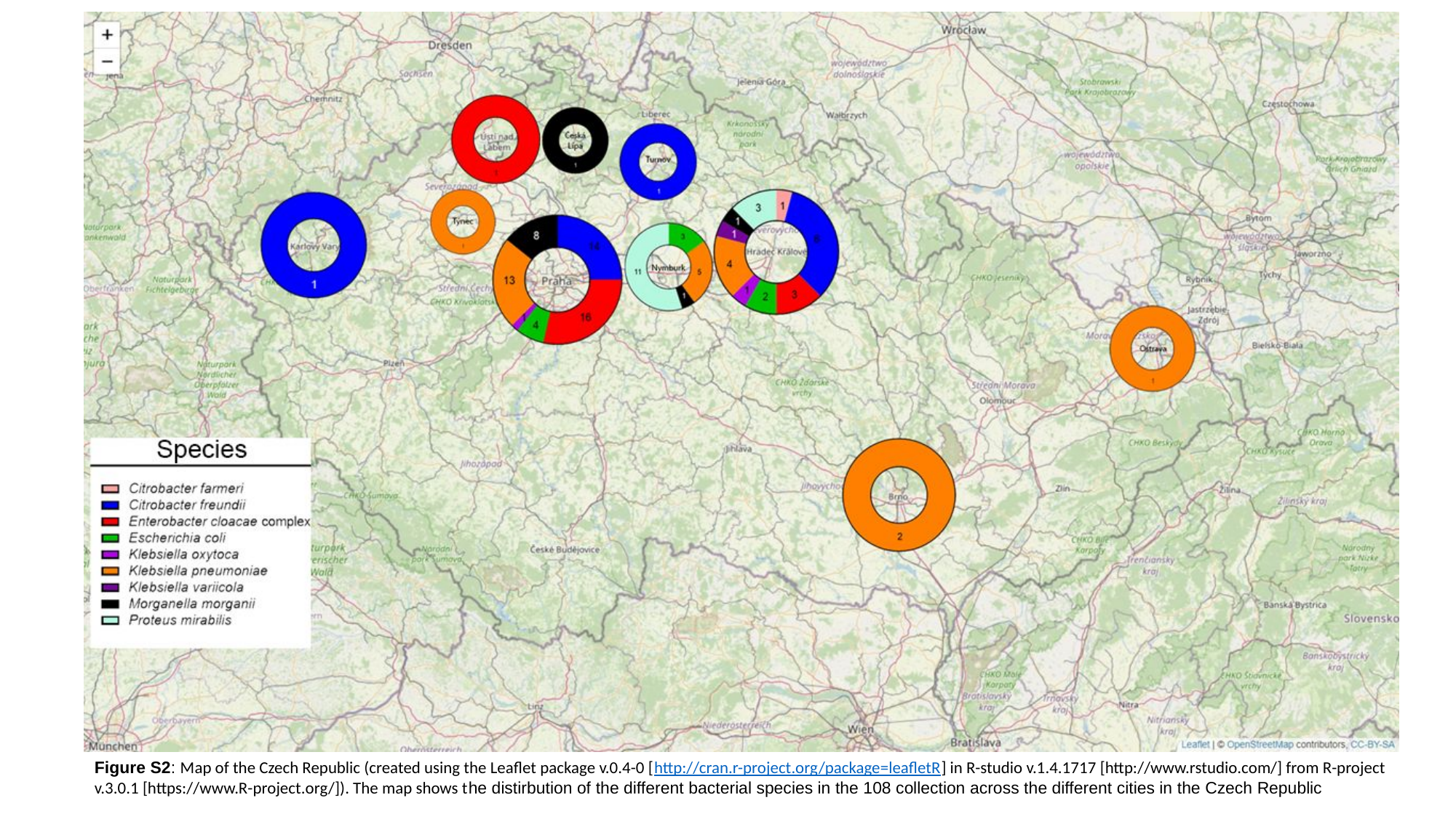

Figure S2: Map of the Czech Republic (created using the Leaflet package v.0.4-0 [http://cran.r-project.org/package=leafletR] in R-studio v.1.4.1717 [http://www.rstudio.com/] from R-project v.3.0.1 [https://www.R-project.org/]). The map shows the distirbution of the different bacterial species in the 108 collection across the different cities in the Czech Republic
