# Supplementary material for: Evidence of an epidemic spread of KPC-producing Enterobacterales in Czech hospitals
Source: Sci Rep. 2021 Aug 3;11:15732. doi: 10.1038/s41598-021-95285-z (PMC8333104; doi:10.1038/s41598-021-95285-z)
Supplement: Supplementary file 5 — Supplementary Information 5. [file 41598_2021_95285_MOESM5_ESM.pptx]

## Slide 1
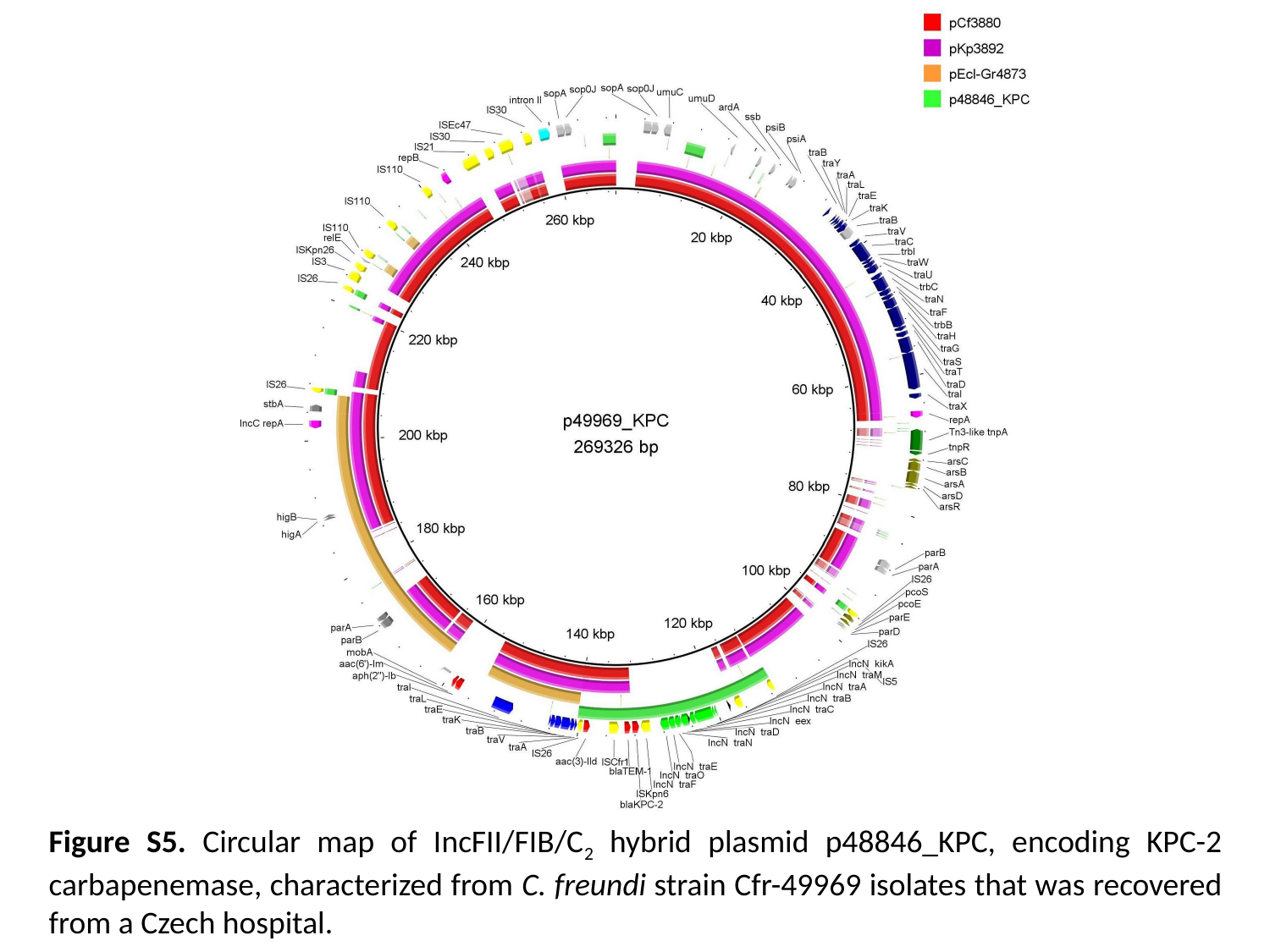

Figure S5. Circular map of IncFII/FIB/C2 hybrid plasmid p48846_KPC, encoding KPC-2 carbapenemase, characterized from C. freundi strain Cfr-49969 isolates that was recovered from a Czech hospital.
