# Supplementary material for: Evidence of an epidemic spread of KPC-producing Enterobacterales in Czech hospitals
Source: Sci Rep. 2021 Aug 3;11:15732. doi: 10.1038/s41598-021-95285-z (PMC8333104; doi:10.1038/s41598-021-95285-z)
Supplement: Supplementary file 6 — Supplementary Information 6. [file 41598_2021_95285_MOESM6_ESM.pptx]

## Slide 1
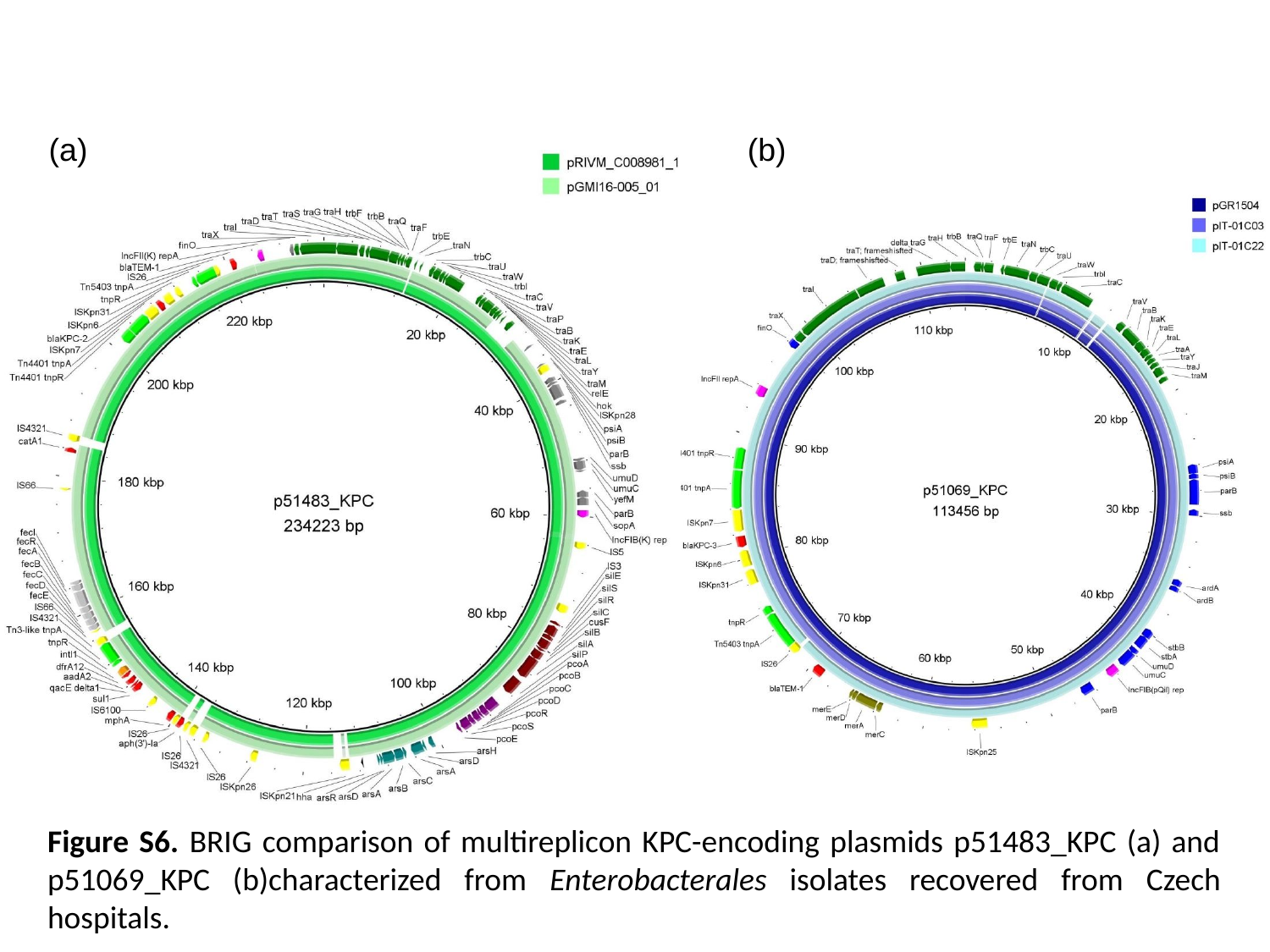

(a)
(b)
Figure S6. BRIG comparison of multireplicon KPC-encoding plasmids p51483_KPC (a) and p51069_KPC (b)characterized from Enterobacterales isolates recovered from Czech hospitals.
